# Seroepidemiological assessment of SARS-CoV-2 vaccine responsiveness and associated factors in the vaccinated community of the Casablanca-Settat Region, Morocco

**DOI:** 10.1038/s41598-024-58498-6

**Published:** 2024-04-03

**Authors:** Sayeh Ezzikouri, Raji Tajudeen, Hind Majidi, Soad Redwane, Safaa Aqillouch, Mohammed Abdulaziz, Merawi Aragaw, Mosoka Papa Fallah, Senga Sembuche, Serge Batcho, Patrick Kabwe, Elizabeth Gonese, Oumaima Laazaazia, Mohcine Elmessaoudi-Idrissi, Nadia Meziane, Abdelhakim Ainahi, M’hammed Sarih, Ahmed E. Ogwell Ouma, Abderrahmane Maaroufi

**Affiliations:** 1 Virology Unit, Viral Hepatitis Laboratory, Institut Pasteur du Maroc, 1 Place Louis Pasteur, 20360 Casablanca, Morocco; 2 Africa Centres for Disease Control and Prevention, African Union, Addis Ababa, Ethiopia; 3 Ministry of Health and Social Protection, Rabat, Morocco; 4 Direction Régionale de la santé Casablanca-Settat, Observatoire régional de santé, Casablanca, Morocco; 5 Centre Régional de Transfusion Sanguine, Casablanca, Morocco; 6 Hormonology and Tumor Markers Laboratory, Institut Pasteur du Maroc, Casablanca, Morocco; 7 Service de Parasitologie et des Maladies Vectorielles, Institut Pasteur du Maroc, Casablanca, Morocco

**Keywords:** Herd immunity, Hybrid immunity, Vaccination, COVID-19, Humoral response, Morocco, North Africa, Vaccines, Immunology, Humoral immunity

## Abstract

Assessing the prevalence of SARS-CoV-2 IgG positivity through population-based serological surveys is crucial for monitoring COVID-19 vaccination efforts. In this study, we evaluated SARS-CoV-2 IgG positivity within a provincial cohort to understand the magnitude of the humoral response against the SARS-CoV-2 vaccine and to inform evidence-based public health decisions. A community-based cross-sectional seroprevalence study was conducted, involving 10,669 participants who received various vaccines (two doses for BBIBP-CorV/Sinopharm, Covishield vaccine, and Pfizer/BioNTech, and one dose for Johnson & Johnson's Janssen COVID-19 vaccine). The study spanned 16 provinces in the Casablanca-Settat region from February to June 2022, during which comprehensive demographic and comorbidity data were collected. We screened samples for the presence of IgG antibodies using the SARS-CoV-2 IgG II Quant assay, which quantifies antibodies against the receptor-binding domain (RBD) of the spike (S) protein, measured on the Abbott Architect i2000SR. The overall crude seroprevalence was 96% (95% CI: 95.6–96.3%), and after adjustment for assay performance, it was estimated as 96.2% (95% CI: 95.7–96.6). The adjusted overall seroprevalences according to vaccine brands showed no significant difference (96% for BBIBP-CorV/Sinopharm, 97% for ChAdOx1 nCoV-19/Oxford/AstraZeneca, 98.5% for BNT162b2/Pfizer-BioNTech, and 98% for Janssen) (*p* = 0.099). Participants of older age, female sex, those with a history of previous COVID-19 infection, and those with certain chronic diseases were more likely to be seropositive among ChAdOx1 nCoV-19/Oxford/AstraZeneca and BBIBP-CorV/Sinopharm vaccinee groups. Median RBD antibody concentrations were 2355 AU/mL, 3714 AU/mL, 5838 AU/mL, and 2495 AU/mL, respectively, after two doses of BBIBP-CorV/Sinopharm, ChAdOx1 nCoV-19/Oxford/AstraZeneca, BNT162b2/Pfizer-BioNTech, and after one dose of Janssen (*p* < 0.0001). Furthermore, we observed that participants vaccinated with ChAdOx1 nCoV-19/Oxford/AstraZeneca and BBIBP-CorV/Sinopharm with comorbid chronic diseases exhibited a more pronounced response to vaccination compared to those without comorbidities. In contrast, no significant differences were observed among Pfizer-vaccinated participants (*p* > 0.05). In conclusion, our serosurvey findings indicate that all four investigated vaccines provide a robust humoral immune response in the majority of participants (more than 96% of participants had antibodies against SARS-CoV-2). The BNT162b2 vaccine was found to be effective in eliciting a strong humoral response compared to the other three vaccines. However, challenges still remain in examining the dynamics and durability of immunoprotection in the Moroccan context.

## Introduction

Severe acute respiratory syndrome coronavirus 2 (SARS-CoV-2)—the causative agent of the coronavirus disease 2019 (COVID-19)—emerged since 2019^1^, and is now posing further challenges in many countries worldwide as new variants are identified.

As of March 29, 2023, there have been 761,402,282 confirmed COVID-19 cases and 6,887,000 deaths globally (WHO Coronavirus (COVID-19) Dashboard) (https://covid19.who.int/). Vaccines are crucial in the fight against SARS-CoV-2, with a total of 13,331,975,343 doses administered as of March 28, 2023 (WHO Coronavirus (COVID-19) Dashboard) (https://covid19.who.int/). In this context, the Moroccan Directorate of Medicine and Pharmacy has authorized four COVID-19 vaccines for emergency use: BBIBP-CorV (Sinopharm, the Beijing Institute of Biological Products, China), BNT 162b2 (Comirnaty, Pfizer, USA), AZD1222 (ChAdOx1 nCoV-19, COVISHIELD, Oxford University-AstraZeneca, UK), and Jcovden (Ad26.COV2.S, formerly known as Janssen COVID-19 vaccine, Janssen Biotech, Inc., USA).

On January 28th, 2021, the kingdom launched the national vaccination campaign based on short-term randomized controlled trials that have shown efficacy against symptomatic disease ranging from 62–96% after the second dose^2–7^. As of April 21, 2023, 23,423,963 individuals have received their second dose (vaccination coverage rate approximately 65% of the population). Additionally, 6,883,240 participants have received their third dose, and 60,739 individuals are vaccinated with the fourth dose of a COVID-19 vaccine (http://www.covidmaroc.ma/Pages/Accueilfr.aspx). Morocco has achieved the highest vaccination coverage among African countries. While the published trial efficacy data to date seem to justify the emergency use of COVID-19 vaccines in Morocco, it is important to conduct further studies among vaccinated populations. Serological studies remain a useful tool to evaluate the implementation of vaccination campaigns and monitor population-level immune responses over time, considering contextual factors specific to our country, such as genetic specificities, co-morbidities, organization of the supply and dispensation system, as well as the duration of the immunity conferred^8,9^. Moreover, the emergence of viral variants that have acquired mutations in the S gene highlights the need for ongoing characterization of vaccine-induced immunity^10,11^.

As no SARS-CoV-2-related serological data from Morocco are available so far, the actual prevalence of antibodies induced by infection and/or vaccination remains unclear. To address this gap, we conducted a community-based cross-sectional seroprevalence study following a COVID-19 mass vaccination campaign in the Casablanca-Settat region, Morocco, during February-June 2022. Additionally, our aim was to characterize the humoral response according to vaccine type, sociodemographic data, comorbid medical conditions, and previous SARS-CoV-2 infection.

## Materials and methods

### Study design, participants and settings

This cross-sectional seroepidemiological study encompassed 10,669 individuals aged 14 years and above. Conducted in 16 provinces of the Casablanca-Settat region from February 1, 2022, to June 27, 2022, the study focused on individuals fully vaccinated with various COVID-19 vaccines (Two doses for Covishield vaccine/ChAdOx1 nCoV-19 Corona Virus Vaccine; Recombinant), BBIBP-CorV/Sinopharm, and Pfizer/BioNTech and one dose for JCovden/Johnson & Johnson's COVID-19 vaccine).

Within each province, a purposive selection was made of public primary health care centers. In each health center, a local leader (a physician) and an investigator (nurse) were assigned to conduct the survey. Participants included consenting individuals visiting the public primary health care centers or blood donors at the transfusion center during the survey period.

Each subject was informed about the study before being sampled and comprehensive demographic and comorbidities data were completed. In accordance with general data protection rules, subject details were kept confidential and after sample collection, full names were removed and were replaced with participant codes. The study protocol was in accordance with the Helsinki declaration and received an approval from the ethics committee of Ibn Rochd hospital in Casablanca (N°01/22) and written informed consent was obtained from each participant.

A staff member conducted face-to-face interviews with consenting individuals using a well-designed questionnaire, which was then completed on a computer. Each participant assigned a unique eleven‐digit identification number. The questionnaire aimed to gather socio-demographic information and potential risk factors for SARS-CoV-2 infection. Variables encompassed self-reported sociodemographic data, along with conditions such as hypertension, diabetes, respiratory disease, smoking, alcohol intake, drug addiction, cancer, cardiovascular disease, chronic renal failure, neurological disease, mental health conditions, systemic disease, infectious syndrome, metabolic syndrome, history of prior COVID-19, and details about COVID-19 vaccination types.

The data collected through the questionnaire were subsequently integrated with the results of laboratory antibody tests using Microsoft Excel 2021 software.

Following the questionnaire completion, a nurse utilized an aseptic procedure to collect 4 ml of EDTA blood samples from consenting participants. The collected samples were promptly transferred to the COVID-19 serology laboratory at the Pasteur Institute of Morocco. Upon arrival at the laboratory, the samples underwent centrifugation at 2500 rpm for 15 min.

The exclusion criterion encompassed any participant experiencing a decline in willingness post-consent or reporting contraindications to venepuncture.

### Serological assay

Serological tests aimed at assessing immune status and monitoring antibody response in individuals who received the COVID-19 vaccine were conducted at the Viral Hepatitis Laboratory of the Pasteur Institute of Morocco. Given that the receptor-binding domain (RBD) serves as the principal immunodominant determinant of the trimeric surface spike glycoprotein, immune responses against RBD are considered the most reliable indicators of both past infection and vaccine-induced immunity^10,11^. We employed the SARS-CoV-2 IgG II Quant assay, a chemiluminescent microparticle immunoassay (CMIA), for both qualitative and quantitative determination of IgG antibodies specific to the receptor-binding domain (RBD) of the S1 subunit of the spike protein of SARS-CoV-2 in human plasma. This assay was conducted using the Abbott Architect i2000SR instrument manufactured by Abbott Laboratories, based in Abbott Park, Illinois.

Results were interpreted according to the manufacturer's criteria: negative if the cutoff value was < 50 AU/ml and positive if the cutoff value was ≥ 50 AU/ml. The assay is characterized by a documented sensitivity of 91.6% and specificity of 99.4%, which is deemed acceptable for conducting SARS-CoV-2 seroprevalence surveys, as previously reported^12^. The analytical measurement interval for the SARS-CoV-2 IgG II Quant assay is specified as ranging from 9.9 to 40,000 AU/ml.

### Statistical analysis

Descriptive statistics were presented in terms of frequency and percentage for categorical variables, while continuous variables were expressed as median and interquartile range (IQR). Both crude and adjusted seroprevalences were reported as proportions with corresponding 95% confidence intervals (CI). The sensitivity and specificity values, namely 91.6% and 99.4% respectively, were calculated based on a prior assessment described in the serological assay^12^. Bayes rule applied to estimate the adjusted seroprevalence (π) as a function of crude seroprevalence (p), sensitivity (r), and specificity (s) according to the formula: π = [p − (1 − s)/r − (1 − s)] as reported previously^13,14^.

The chi-square test was employed to examine associations among categorical variables. Univariate and multivariate logistic regression models were employed to estimate odds ratios (ORs), considering IgG antibody status as the outcome variable. Independent variables included sex (male/female), age, hypertension, diabetes, respiratory disease, cancer, cardiovascular diseases, chronic kidney disease, physical distancing, mask-wearing behavior, and past history of confirmed COVID-19 disease (present/absent). Variables with a *p*-value < 0.05 in the univariate analysis were subsequently included in the multivariate analysis.

Mann–Whitney U Test and Kruskal–Wallis test were employed for comparing nonparametric continuous variables across different groups. Statistical analyses were carried out using R software for Windows and GraphPad PRISM version 6e (GraphPad Software, San Diego, CA, USA). A *p*-value less than 0.05 was considered statistically significant, and two-tailed comparisons were performed.

### Ethics approval

This study was conducted in accordance with the Declaration of Helsinki. The study was approved by the ethics committee of Ibn Rochd hospital in Casablanca (N°01/22).

## Results

### Basic characteristics of the study participants and overall seroprevalence among the vaccinated community

The distribution of participants based on socio-demographic data and potential risk factors for SARS-CoV-2 infection is presented in Table 1. Out of the total, 10,669 blood samples were assessed (12% of participants refused to participate in the serological survey), comprising 7,380 women and 3,475 men, with a median age of 44 years (ranging from 14 to 102 years) (Table 1).

**Table 1:** Participants characteristics.

	All No. (%)	Women No. (%)	Men No. (%)
Gender	10,669 (100.0)	7380 (69.2)	3289 (30.8)
Age groups (years)			
14–19	351 (3.3)	255 (3.5)	96 (2.9)
20–49	6081 (57.0)	4056 (55.0)	2025 (61.6)
50–64	2909 (27.3)	2133 (28.9)	776 (23.6)
≥ 65	1328 (12.4)	936 (12.7)	392 (11.9)
Self-reported hypertension			
No	9349 (87.6)	6332 (85.8)	3017 (91.7)
Yes	1320 (12.4)	1048 (14.2)	272 (8.3)
Self-reported diabetes			
No	9195 (86.2)	6222 (84.3)	2973 (90.4)
Yes	1474 (13.8)	1158 (15.7)	316 (9.6)
Self-reported cardiovascular disease			
No	10,427 (97.7)	7184 (97.3)	3243 (98.6)
Yes	242 (2.3)	196 (2.7)	46 (1.4)
Self-reported respiratory diseases			
No	10,348 (97.0)	7119 (96.5)	3229 (98.2)
Yes	321 (3.0)	261 (3.5)	60 (1.8)
Self-reported cancer			
No	10,627 (99.6)	7345 (99.5)	3282 (99.8)
Yes	42 (0.4)	35 (0.5)	7 (0.2)
Self-reported chronic kidney disease			
No	10,105 (94.7)	7082 (96.0)	3023 (91.9)
Yes	564 (5.3)	298 (4.0)	266 (8.1)
Self-reported physical distancing*			
No	8542 (80.3)	5830 (79.4)	2712 (82.5)
Yes	2089 (19.7)	1515 (20.6)	574 (17.5)
Self-reported mask-wearing behavior*			
No	4840 (45.5)	2992 (40.7)	1848 (56.3)
Yes	5790 (54.5)	4353 (59.3)	1437 (43.7)
Self-reported PCR-confirmed diagnosis*			
No	8661 (82.6)	5978 (81.8)	2683 (84.3)
Yes	1825 (17.4)	1326 (18.2)	499 (15.7)

*Data are missing.

The serological survey encompassed 10,669 participants, with 8,602 having received two doses of BBIBP-CorV/Sinopharm (mean ± SEM of months after the second dose = 8.11 ± 0.08), 1,817 with ChAdOx1 nCoV-19 (COVISHIELD, Oxford/AstraZeneca; mean ± SEM of months after the second dose = 9.82 ± 0.15), 208 with BNT162b2 (Pfizer-BioNTech, Comirnaty, Pfizer, USA; mean ± SEM of months after the second dose = 7.14 ± 0.55), and 42 participants vaccinated with a single dose of JCovden/Johnson & Johnson's COVID-19 vaccine (mean ± SEM of months after the first dose = 7.50 ± 1.00). Adjusted overall seroprevalences did not show significant differences between vaccines (*p* = 0.099).

### Seroprevalence in the BBIBP-CorV (Sinopharm)-vaccinated community

We determined an adjusted seroprevalence rate of 96% (95% CI: 95.5% to 96.4%) among participants who received the BBIBP-CorV vaccine (Table 2). Logistic regression analysis revealed several variables associated with a higher risk of detecting anti-SRAS-CoV-2 antibodies (Table 3). Specifically, a higher prevalence of antibody positivity was linked to age. Additionally, the odds of being antibody-positive are 0.59 times lower for male participants than for females. A higher prevalence of antibody positivity was associated with hypertension, mask-wearing, and PCR-confirmed COVID-19 disease (Table 3). Furthermore, the analysis by chronic kidney disease revealed that participants with kidney disease had a significantly lower probability of being seropositive than participants without kidney disease (Table 3).

**Table 2:** Composition of the cohort with respect to vaccine type.

Vaccine types	Participants No. (%)	Women No. (%)	Men No. (%)	Anti-RBD positive No. (%)	Crude seroprevalence (%), (95% CI)	Adjusted seroprevalence* (%), (95% CI)
BBIBP–CorV (Sinopharm)	8602 (80.6)	5824 (67.7)	2778 (32.3)	8236 (95.7)	95.7 (95.3–96.2)	96.0 (95.5–96.4)
ChAdOx1 nCoV–19 (COVISHIELD)	1817 (17.0)	1394 (76.7)	423 (23.3)	1757 (96.7)	96.7 (95.7–97.4)	97.0 (96.0–97.9)
BNT162b2 (Pfizer–BioNTech, cominarty)	208 (1.9)	138 (66.3)	70 (33.7)	204 (98.1)	98.1 (94.8–99.4)	98.5 (95.0–100.0)
Johnson and Johnson's Janssen	42 (0.4)	24 (57.1)	18 (42.9)	41 (97.6)	97.6 (85.9–99.9)	98.0 (85.2–100.0)
Total	10,669 (100.0)	7380 (69.2)	3289 (30.8)	10,238 (96.0%)	96.0 (95.6–96.3)	96.2 (95.7–96.6)

*Adjusted seroprevalence based on the assay sensitivity and sepecifity.

**Table 3:** Univariate and multivariate analyses for the association between risk factors and SARS–CoV–2 seropositivity, based on a logistic regression model among 8,602 participants receiving two doses of BBIBP–CorV vaccine from 16 provinces of the region of Casablanca–Settat, Morocco during February to June 2022.

Variables	Anti-RBD negative No. (%)	Anti-RBD positive No. (%)	Univariate analysis		Multivariate analysis	
			OR (95%)	*p* value	OR (95%)	*p* value
Age groups (years)					0.99 (0.98–1.00)	0.039
14–19	5 (1.8)	266 (98.2)	1.00			
20–49	225 (4.3)	5067 (95.7)	0.41 (0.17–1.00)	0.050		
50–64	94 (4.3)	2108 (95.7)	0.41 (0.17–1.01)	0.053		
≥ 65	42 (5.1)	786 (94.9)	0.34 (0.13–0.87)	0.024		
Sex						
Women	204 (3.5)	5620 (96.5)	1.00			
Men	162 (5.8)	2616 (94.2)	0.59 (0.47–0.72)	< 0.001	0.64 (0.44–0.95)	0.00009
Hypertension						
No	345 (4.5)	7372 (95.5)	1.00			
Yes	21 (2.4)	864 (97.6)	1.92 (1.23–3.00)	0.004	2.05 (1.30–3.30)	0.003
Diabetes						
No	333 (4.4)	7249 (95.6)	1.00			
Yes	33 (3.2)	987 (96.8)	1.37 (0.95–1.98)	0.087		
Cardiovascular disease						
No	356 (4.2)	8086 (95.8)	1.00			
Yes	10 (6.3)	150 (93.7)	0.66 (0.34–1.26)	0.210		
Respiratory diseases						
No	359 (4.3)	8002 (95.7)	1.00			
Yes	7 (2.9)	234 (97.1)	1.50 (0.70–3.20)	0.295		
Cancer						
No	366 (4.3)	8205 (95.7)	1.00			
Yes	0 (0.0)	2 (100.0)	–	–		
Chronic kidney disease						
No	332 (4.1)	7766 (95.9)	1.00			
Yes	34 (6.7)	470 (93.3)	0.59 (0.41–0.85)	0.005	0.64 (0.44–0.95)	0.022
Physical distancing						
No	293 (4.3)	6574 (95.7)	1.00			
Yes	73 (4.3)	1624 (95.7)	0.99 (0.76–1.29)	0.949		
Mask-wearing behavior						
No	206 (4.9)	3964 (95.1)	1.00			
Yes	160 (3.6)	4234 (96.4)	1.37 (1.11–1.70)	0.003	1.27 (0.91–1.58)	0.031
PCR-confirmed diagnosis*						
No	325 (4.6)	6730 (95.4)	1.00			
Yes	39 (2.7)	1400 (97.3)	1.73 (1.24–2.43)	0.001	1.71 (1.23–2.44)	0.002

*Data are missing.

### Seroprevalence in the Covishield/AstraZeneca vaccinated community

For participants vaccinated with ChAdOx1-nCov-19, the adjusted seroprevalence was 97% (95% CI: 96–97.9) (Table 2). Logistic regression analysis assessing the association between SARS-CoV-2 seropositivity and demographic characteristics and chronic diseases between February and June 2022 is presented in Table 4. In univariable analysis, participants in the age groups of more than 14–19 years and those with a PCR-confirmed diagnosis (OR = 2.65; 95% CI: 1.05–6.68; *p* = 0.038) tended to be significantly more likely to be seropositive for anti-SARS-CoV-2 antibodies (Table 4). In contrast, in the multivariable analysis, only participants with a PCR-confirmed diagnosis were more likely to be seropositive for anti-RBD (OR = 2.69; 95% CI: 1.17–7.78; *p* = 0.036) (Table 4).

**Table 4:** Univariate and multivariate analyses for the association between risk factors and SARS–CoV–2 seropositivity, based on a logistic regression model among 1,817 participants receiving two doses of ChAdOx1nCoV–19 vaccine from 16 provinces of the region of Casablanca–Settat, Morocco during February to June 2022.

Variables	Anti-RBD negative No. (%)	Anti-RBD positive No. (%)	Univariate analysis		Multivariate analysis	
			OR (95%)	*p* value	OR (95%)	*p* value
Age groups (years)					1.00 (0.99–1.02)	0.734
14–19	4 (13.8)	25 (86.2)	1.00			
20–49	17 (2.7)	602 (97.3)	5.67 (1.78–18.08)	0.003		
50–64	22 (3.2)	655 (96.8)	4.76 (1.53–14.86)	0.007		
≥ 65	17 (3.5)	475 (96.5)	4.47 (1.40–14.28)	0.011		
Sex						
Women	42 (3.0)	1352 (97.0)	1.00			
Men	18 (4.3)	405 (95.7)	0.70 (0.40–1.23)	0.213		
Hypertension						
No	43 (3.1)	1344 (96.9)	1.00			
Yes	17 (4.0)	413 (96.0)	0.78 (0.44–1.38)	0.388		
Diabetes						
No	42 (3.0)	1339 (97.0)	1.00			
Yes	18 (4.1)	418 (95.9)	0.73 (0.41–1.28)	0.270		
Cardiovascular disease						
No	57 (3.3)	1681 (96.7)	1.00			
Yes	3 (3.8)	76 (96.2)	0.86 (0.26–2.80)	0.801		
Respiratory diseases						
No	59 (3.4)	1685 (96.6)	1.00			
Yes	1 (1.4)	72 (98.6)	2.52 (0.34–18.43)	0.362		
Cancer						
No	60 (3.3)	1746 (96.7)	1.00			
Yes	0 (0.0)	11 (100.0)	–	–		
Chronic kidney disease						
No	59 (3.4)	1701 (96.6)	1.00			
Yes	1 (1.8)	56 (98.2)	1.94 (0.26–14.27)	0.514		
Physical distancing						
No	50 (3.4)	1429 (96.6)	1.00			
Yes	10 (3.0)	328 (97.0)	1.15 (0.58–2.29)	0.695		
Mask-wearing behavior						
No	15 (2.6)	567 (97.4)	1.00			
Yes	45 (3.6)	1190 (96.4)	0.70 (0.39–1.27)	0.238		
PCR-confirmed diagnosis*						
No	54 (3.7)	1397 (96.3)	1.00			
Yes	5 (1.4)	343 (98.6)	2.65 (1.05–6.68)	0.038	2.69 (1.17–7.78)	0.036

*Data are missing.

### Seroprevalence in the BNT162b2/Pfizer-BioNTech vaccination community

For participants vaccinated with BNT162b2, the overall adjusted prevalence of anti-SRAS-CoV-2 antibodies was 98.5% (95% CI: 95.0–100.0) (Table 2). Logistic regression analysis showed that participants with hypertension who received BNT162b2 were more likely to be seronegative compared to female participants and those without hypertension (Table 5).

**Table 5:** Univariate and multivariate analyses for the association between risk factors and SARS–CoV–2 seropositivity, based on a logistic regression model among 208 participants receiving two doses of BNT162b2 vaccine from 16 provinces of the region of Casablanca–Settat, Morocco during February to June 2022.

Variables	Anti-RBD negative No. (%)	Anti-RBD positive No. (%)	Univariate analysis		Multivariate analysis	
			OR (95%)	*p* value	OR (95%)	*p* value
Age groups (years)						
14–19	0 (0.0)	39 (100.0)	1.00			
20–49	3 (2.3)	129 (97.7)	–	–		
50–64	1 (3.8)	25 (96.2)	–	–		
≥ 65	0 (0.0)	8 (100.0)	–	–		
Sex						
Women	0 (0.0)	138 (100.0)	1.00			
Men	4 (5.7)	66 (94.3)	–	–		
Hypertension						
No	3 (1.5)	201 (98.5)	1.00			
Yes	1 (25.0)	3 (75.0)	0.04 (0.004–0.56)	0.016	0.03 (0.002–1.48)	0.02
Diabetes						
No	4 (2.1)	186 (97.9)	1.00			
Yes	0 (0.0)	18 (100.0)	–	–		
Cardiovascular disease						
No	4 (1.9)	202 (98.1)	1.00			
Yes	0 (0.0)	2 (100.0)	–	–		
Respiratory diseases						
No	4 (2.0)	199 (98.0)	1.00			
Yes	0 (0.0)	5 (100.0)	–	0.377		
Cancer						
No	4 (1.9)	204 (98.1)	1.00			
Yes	0 (0.0)	0 (0.0)	–	–		
Chronic kidney disease						
No	4 (1.9)	202 (98.1)	1.00			
Yes	0 (0.0)	2 (100.0)	–	–		
Physical distancing						
No	4 (2.6)	151 (97.4)	1.00			
Yes	0 (0.0)	53 (100.0)	–	–		
Mask-wearing behavior*						
No	1 (1.7)	58 (98.3)	1.00			
Yes	2 (1.4)	146 (98.6)	1.26 (0.11–14.15)	0.852		
PCR-confirmed diagnosis*						
No	4 (2.5)	159 (97.5)	1.00			
Yes	0 (0.0)	39 (100.0)	–	–		

*Data are missing.

### Seroprevalence in the JCovden-vaccinated community

The adjusted seroprevalence among participants vaccinated with the Janssen/Johnson & Johnson's COVID-19 vaccine was 98% (95% CI: 85.2–100.0). Owing to the small sample size (n = 42), logistic regression analysis of the association between SARS-CoV-2 seropositivity and demographic characteristics and comorbid conditions was not conducted.

### Anti-SARS-CoV-2 IgG antibodies induced by vaccination

**Figure 1:**
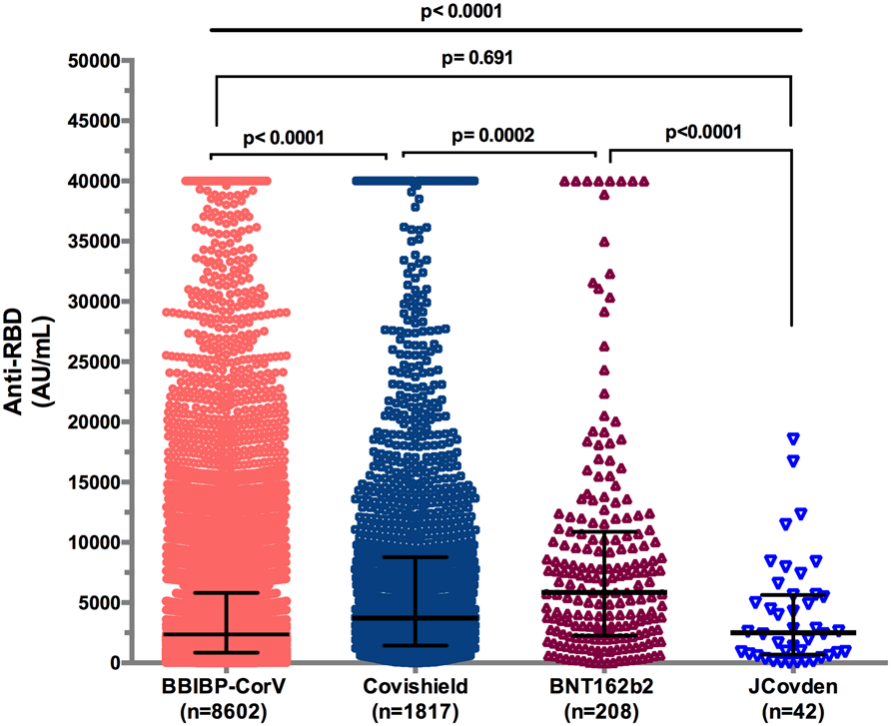
Anti-SARS-CoV-2 antibody levels among four vaccine brands. Data are presented as median and interquartile range for IgG antibody. Mann–Whitney and Kruskal–Wallis tests were used.

Subsequently, we evaluated the magnitude of the humoral response by measuring IgG antibodies to the RBD of the S1 subunit of the SARS-CoV-2 spike protein. The median RBD antibody concentrations were 2355 AU/mL, 3714 AU/mL, 5838 AU/mL, and 2495 AU/mL after two doses of BBIBP-CorV/Sinopharm, ChAdOx1 nCoV-19/Oxford/AstraZeneca, BNT162b2/Pfizer-BioNTech, and after one dose of JCovden/Johnson & Johnson's COVID-19 vaccine. Significant differences were observed among vaccine brands (*p* < 0.0001). Notably, there was no significant difference between the JCovden vaccine and the BBIBP-CorV vaccine (*p* = 0.691) (Fig. 1).

**Figure 2:**
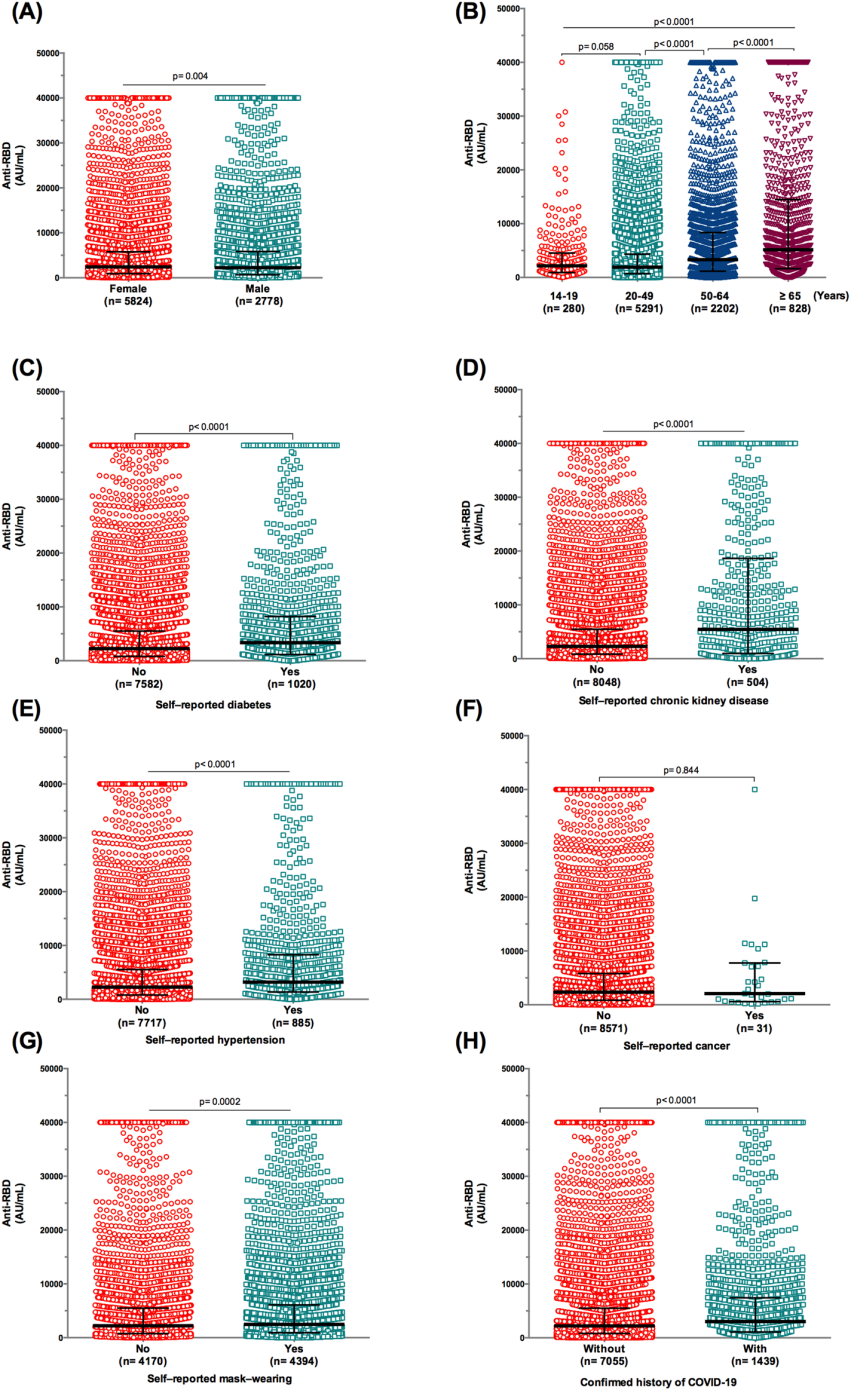
Anti-RBD IgG antibody responses to BBIBP-CorV vaccine/Sinopharm in the general population. (**A**) Antibody levels subdivided by gender. (**B**) Anti-SARS-CoV-2 IgG levels by age. (**C**) SARS-CoV-2 antibody titer in participants with and without diabetes. (**D**) SARS-CoV-2 antibody titer in participants with and without chronic kidney disease. (**E**) SARS-CoV-2 antibody titer in participants with and without hypertension. (**F**) SARS-CoV-2 antibody titer in participants with and without cancer. (**G**) SARS-CoV-2 antibody titer in participants with and without mask-wearing. (**H**) Antibody levels by the history of coronavirus disease 2019 (COVID-19). Data are presented as median and interquartile range for IgG antibody titers. Mann–Whitney test was used for comparisons.

Stratifying participants who received the BBIBP-CorV vaccine revealed a significant difference in antibody concentration titers by gender (median = 2428 vs. 2238 AU/mL for females and males, respectively) (*p* = 0.004) (Fig. 2A). An association was identified between age and anti-RBD IgG levels in BBIBP-CorV vaccine recipients (< 0.0001), with the highest levels observed in those aged ≥ 65 years (median = 5145.5 AU/mL) (Fig. 2B).

Unexpectedly, participants with comorbidities exhibited the highest levels of anti-RBD IgG (Fig. 2C–E), while for participants with cancer, antibody levels showed no significant difference between those with (median = 2078 AU/mL) and without cancer (median = 2358 AU/mL) (Fig. 2F). Additionally, individuals reporting mask-wearing demonstrated higher anti-RBD antibody titers (median = 2468 AU/mL) compared to those not wearing masks (median = 2215 AU/mL) (*p* = 0.0002) (Fig. 2G).

Lastly, our data revealed that individuals with confirmed exposure to SARS-CoV-2 had elevated anti-RBD antibody titers (median = 3019 AU/mL) compared with uninfected individuals (median = 2222 AU/mL) (Fig. 2H).

**Figure 3:**
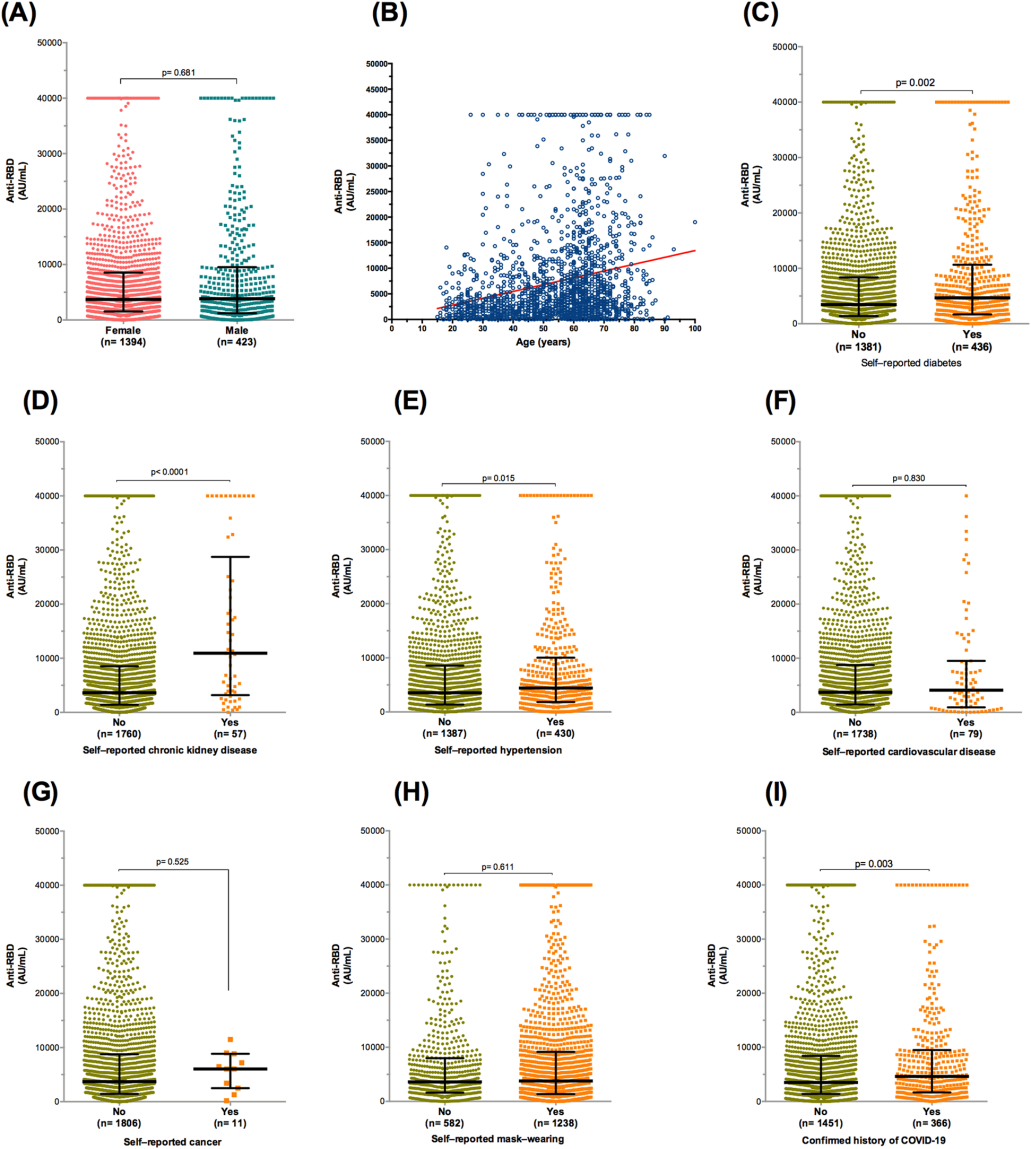
Antibody responses against RBD after two doses of ChAdOx1-nCoV-19/AstraZeneca. (**A**) Gender difference in antibody response. (**B**) Scatter plot of the distribution of antibody titers according to age. (**C**) SARS-CoV-2 antibody titer in participants with and without diabetes. (**D**) SARS-CoV-2 antibody titer in participants with and without chronic kidney disease. (**E**) SARS-CoV-2 antibody titer in participants with and without hypertension. (**F**) SARS-CoV-2 antibody titer in participants with and without cardiovascular disease. (**G**) SARS-CoV-2 antibody titer in participants with and without cancer. (**H**) SARS-CoV-2 antibody titer in participants with and without mask-wearing. (**I**) Antibody levels by the history of coronavirus disease 2019. Data are presented as median and interquartile range for IgG antibody titers. Spearman correlation and Mann–Whitney test were used for comparisons.

Stratifying participants who received the Covishield vaccine revealed no significant difference in the humoral response by gender (median = 3698 vs. 3845 AU/mL for females and males, respectively) (*p* = 0.681) (Fig. 3A). In contrast, bivariate Spearman analysis revealed a positive correlation between age and anti-RBD IgG titers (r = 0.240, 95% CI: 0.195 to 0.284, *p* < 0.0001) (Fig. 3B).

Comparison between participants according to medical comorbidities revealed elevated anti-RBD antibody concentrations in those with diabetes (median = 4660.5 vs. 3461 AU/mL for with diabetes and without diabetes, respectively) (*p* = 0.002) (Fig. 3C), chronic hypertension (median = 4416 vs. 3546 AU/mL for with chronic hypertension and without chronic hypertension, respectively) (*p* = 0.015) (Fig. 3D), and renal disease (median = 10,933 vs. 3637 AU/mL for with renal disease and without renal disease, respectively) (*p* < 0.0001) (Fig. 3E). In contrast, there was no statistical difference in antibody titers between participants with cancer (median = 6038 AU/mL) and those without cancer (median = 3708.5 AU/mL) (*p* = 0.525) (Fig. 3F) and cardiovascular disease (median = 4094 vs. 3701 AU/mL for with cardiovascular disease and without cardiovascular disease, respectively) (*p* = 0.830) (Fig. 3G). Additionally, wearing a mask did not affect the humoral response in Covishield-vaccinated participants (Fig. 3H) (*p* = 0.611). However, prior exposure to COVID-19 increased the level of anti-RBD IgG (median = 4618 vs. 3508 AU/mL for individuals with confirmed exposure to SARS-CoV-2 and uninfected individuals, respectively) (*p* = 0.003) (Fig. 3I).

**Figure 4:**
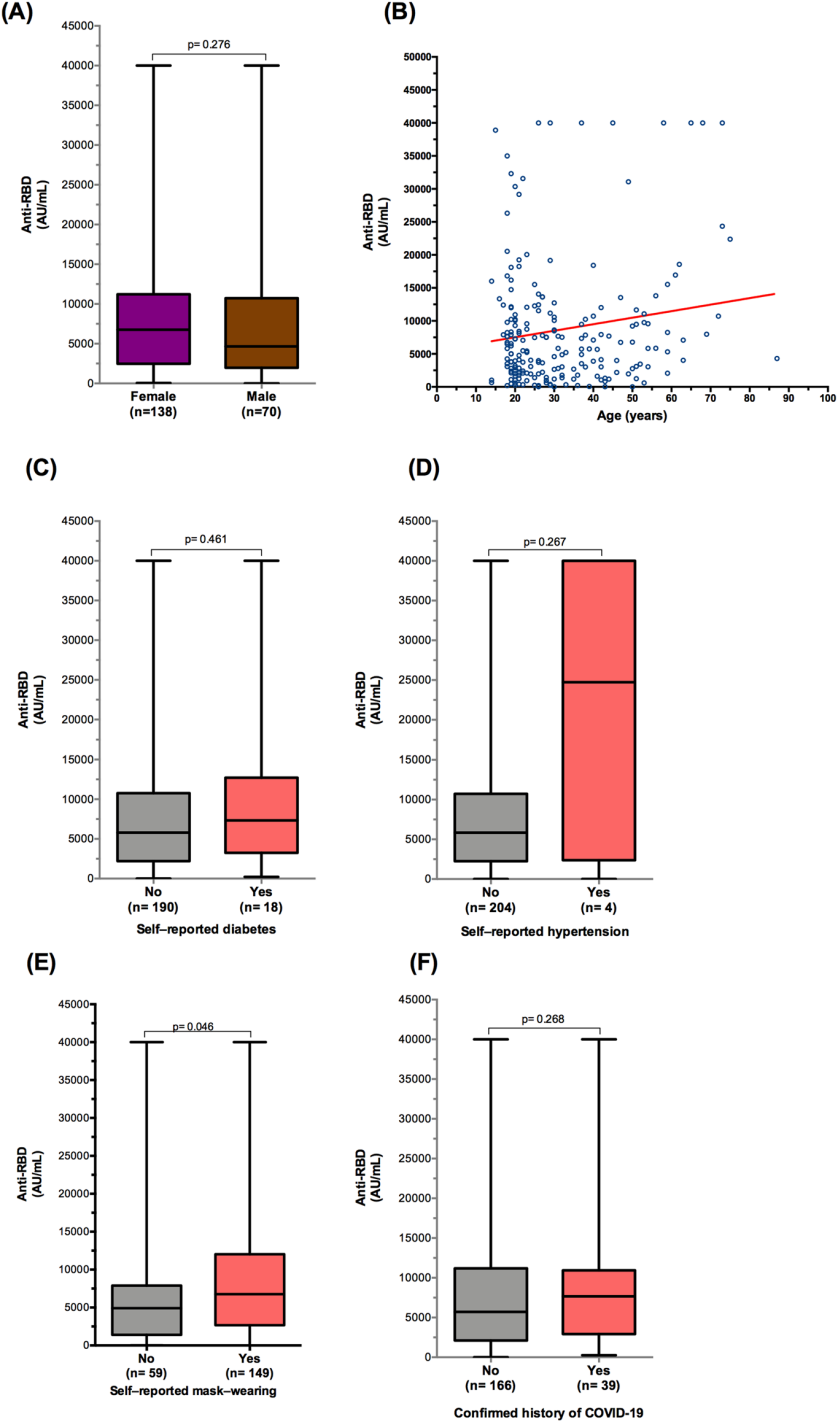
Antibody responses against RBD following two doses of BioNTech162b2/Pfizer vaccine. (**A**) Distribution of antibody titers according to sex. (**B**) Correlation of age and anti-RBD IgG antibody levels. (**C**) SARS-CoV-2 antibody titer in participants with and without diabetes. (**D**) SARS-CoV-2 antibody titer in participants with and without hypertension. (**E**) SARS-CoV-2 antibody titer in participants with and without mask-wearing. (**F**) Antibody levels by the history of coronavirus disease 2019. Data are presented as box and whisker plots with the minimum and maximum range for IgG antibody titers. Spearman correlation and Mann–Whitney test were used for comparisons.

In fully vaccinated participants with BNT162b2/Pfizer, stratification by demographics, comorbidities, and history of COVID-19 showed no significant differences in anti-RBD antibody concentrations (Fig. 4A–D,F). In contrast, an elevated antibody level was observed in participants who reported wearing a mask (median = 6753 AU/mL) compared with those who did not report wearing a mask (median = 4909 AU/mL) (*p* = 0.046) (Fig. 4E).

Stratification of participants vaccinated with JCovden/Johnson & Johnson's COVID-19 vaccine showed no association by gender (*p* = 0.456), and no correlation between age and anti-RBD antibody levels was noted (*p* = 0.362). However, stratification of participants by comorbidities was not performed due to the limited sample size (n = 42).

## Discussion

To foster herd immunity within the community and mitigate the transmission of the virus, Morocco has initiated a mass vaccination program against SARS-CoV-2. The COVID-19 vaccines approved for use in Morocco have demonstrated positive safety and efficacy profiles^4,15^. The vaccines administered in our country have proven effective in preventing deaths and hospitalizations related to SARS-CoV-2 infection^4^. Certainly, understanding the humoral response after SARS-CoV-2 vaccination plays a crucial role in predicting protection against reinfection. This knowledge is instrumental in devising strategies to limit the pandemic and provides valuable insights for public health decision-makers, enabling them to plan more effective and targeted vaccination programs tailored to different populations^16,17^. In this study, the SARS-CoV-2 IgG II Quant assay was employed to quantify IgG antibodies to the receptor-binding domain (RBD). This assay demonstrated a strong correlation with SARS-CoV-2 protein S-specific neutralizing antibodies. These neutralizing antibodies are considered crucial for protection against the disease, reinforcing the reliability of the chosen assay in assessing the humoral response to SARS-CoV-2^18–20^. Among the study participants, a notable proportion exhibited IgG positive antibodies, with a seroprevalence rate of 96%. This figure surpasses the reported seroprevalence in Moroccan healthcare workers (86.59%, using the Abbott Architect test) documented before the surge of the Omicron variant^21^. The observed increase in the antibody rate was primarily attributed to hybrid immunity, defined as the combination of two doses of a COVID-19 vaccine and at least one SARS-CoV-2 infection either before or after the initiation of vaccination^21–23^. Interestingly, the rate of anti-RBD antibody seroconversion (98.5%) was notably higher with the BNT162b2/Pfizer vaccine compared to other vaccine brands. Remarkably, these findings align with those reported in prior studies^24^. Median anti-RBD antibody concentrations were notably high in individuals vaccinated with mRNA (BNT162b2/Pfizer-BioNTech), followed by vector-type vaccines (ChAdOx1 nCoV-19/Oxford/AstraZeneca and JCovden/Johnson & Johnson's COVID-19 vaccine), and an inactivated whole-virion vaccine (BBIBP-CorV/Sinopharm). These trends align with findings reported in previous studies^24–27^. Among participants in this study, a confirmed history of COVID-19 was found to be strongly associated with anti-RBD antibody titers across different vaccine brands. This association has also been documented by other research groups^22,26,28,29^.

We estimated an adjusted seroprevalence rate among participants vaccinated with BBIBP-CorV, the most widely used vaccine in Morocco, of 96%, which is lower than the seroconversion rate (> 99%) reported in the interim results of phase 3 vaccine trials^30^, yet, it is higher than the seroprevalence reported in the Kyrgyz population (91.6%) vaccinated with Sinopharm^31^. In our investigation, seropositivity rates and antibody concentrations were influenced by age and sex, with women exhibiting higher antibody levels than men. This observation appears to be consistent with previous studies^32^. Moreover, participants over 65 years of age displayed a higher seropositivity rate and humoral response than younger participants, in contrast to findings from some previous studies^32,33^, this discrepancy challenges the conventional notion that the post-vaccination humoral response is likely to be low or non-existent in older participants. Furthermore, we observed that participants vaccinated with BBIBP-CorV/Sinopharm with comorbid chronic diseases had a higher seroconversion rate, and comorbidities did not adversely affect their immune response to COVID-19 vaccines. The most significant finding of this study is the association between comorbidities and a higher antibody response to the Sinopharm vaccine. However, a previous study showed that hypertension, diabetes, and combined diseases did not negatively affect the immune response^34^. Due to a considerable number of participants having a history of exposure to SARS-CoV-2 infection, and considering that individuals with comorbid chronic diseases are more susceptible to COVID-19 and are more likely to be hospitalized for further treatment, the probability of developing severe disease following infection is heightened. This elevated risk is reflected in higher hospitalization and mortality rates^35^. Therefore, the heightened humoral response could be attributed to hybrid immunity, aligning with previous research indicating that advanced age is associated with a higher antibody response, potentially linked to multiple chemokine-induced hyperinflammation in COVID-19 patients^36^.

In those vaccinated with the Covishield/AstraZeneca vaccine, the adjusted seroprevalence was 97%, which is lower than that reported in the previous study and phase 2/3 trial of the ChAdOx1 nCoV-19 vaccine^37,38^, but similar to another report^39^. We observed a significant influence of age, but not gender, on seropositivity, and IgG levels increased with age, consistent with a previous study using the Covishield vaccine^40^.

A variety of comorbidities (hypertension, diabetes, heart, kidney, respiratory diseases, etc.) did not affect the seroconversion rate or have a negative effect on the humoral response after two doses of the Covishield vaccine. These data seem to be consistent with the results of previous serological surveys^15,40^. In contrast, a previous study showed no significant difference in the humoral response between participants with and without comorbidities^37^. On the other hand, IgG concentrations are lower in those with comorbidities than in those without^40^.

In Moroccans fully vaccinated with Comirnaty (BNT162b2/Pfizer-BioNTech), we found an overall seroprevalence of 98.5%, consistent with results from general population trials and seroprevalence surveys in several countries^3,41–46^. We showed that male participants and patients with hypertension who received BNT162b2 were more likely to be IgG-negative for SARS-.

CoV-2. Previous data have shown that male gender and the presence of comorbidities predicted seronegativity for SARS-CoV-2^46–48^. Whereas, in participants who received the BNT162b2/Pfizer vaccine, no correlation was observed between IgG antibody concentration and participant age and sex. These results appear to be in agreement with previous studies^29,46^.

Among the limitations of our study, we can highlight that our sample size was relatively small in the study groups vaccinated with BNT162b2/Pfizer and JCovden vaccines. Additionally, for COVID-19 convalescents, the lack of disease severity classification (from asymptomatic to critical) is noteworthy. Although the humoral response is weak, a robust post-infection cellular response is known to provide long-term protection against SARS-CoV-2 infection^49^. Moreover, it is generally accepted that higher antibody levels and neutralizing antibodies specific to the SARS-CoV-2 spike protein are likely to be protective against the disease. Therefore, it would be of interest to assess the cellular immune response and neutralizing antibodies. Additionally, data collection was conducted through a questionnaire, and there is no means to verify the accuracy of the reported chronic diseases, and their prevalence in the general population; this poses a potential limitation in our investigation. Lastly, the 12–13-year-old age group was not included in our study. Future research endeavors will address this age range in subsequent studies.

## Conclusion

To our knowledge, this study is the largest investigation comparing the humoral response of four types of vaccines deployed in Morocco. The data generated by this cross-sectional serological survey could help inform and improve vaccination strategies. This is the first study in the region to assess not only post-vaccination seroprevalence but also the humoral response generated by the four main types of vaccines implemented by the Ministry of Health in Morocco to produce IgG antibodies against SARS-CoV-2. We suggest that further studies on IgG seroprevalence in individuals who received 3 and 4 doses, as well as monitoring the dynamics of the humoral response prospectively, be conducted in our country.

## Data Availability

The data presented in this study are available upon reseanable request from the corresponding author. The data are not publicly available according to the ethical committee decision on the conduct of this study.
